# Characterisation of an Adult Zebrafish Model for *SDHB*-Associated Phaeochromocytomas and Paragangliomas

**DOI:** 10.3390/ijms25137262

**Published:** 2024-07-01

**Authors:** Jasmijn B. Miltenburg, Marnix Gorissen, Inge van Outersterp, Iris Versteeg, Alex Nowak, Richard J. Rodenburg, Antonius E. van Herwaarden, Andre J. Olthaar, Benno Kusters, Catleen Conrad, Henri J. L. M. Timmers, Margo Dona

**Affiliations:** 1 Department of Internal Medicine, Radboud University Medical Center, 6525AG Nijmegen, The Netherlands; jasmijn.miltenburg@radboudumc.com (J.B.M.);; 2 Department of Plant and Animal Biology, Radboud Institute for Biological and Environmental Sciences, Radboud University, 6525AJ Nijmegen, The Netherlands; 3 Departments of Pediatrics and Genetics, Radboud Center for Mitochondrial Medicine, Translational Metabolic Laboratory, Radboud University Medical Center, 6525AG Nijmegen, The Netherlands; 4 Department of Laboratory Medicine, Radboud University Medical Center, 6525AG Nijmegen, The Netherlands; 5 Department of Pathology, Radboud University Medical Center, 6525AG Nijmegen, The Netherlands; 6 Institute of Clinical Chemistry and Laboratory Medicine, Medical Faculty and University Hospital Carl Gustav Carus, Technische Universität Dresden, 01307 Dresden, Germany

**Keywords:** zebrafish, phaeochromocytoma, paraganglioma, *succinate dehydrogenase subunit B* (*SDHB*)

## Abstract

Phaeochromocytomas and paragangliomas (PPGLs) are rare neuroendocrine tumours arising from chromaffin cells. Pathogenic variants in the gene *succinate dehydrogenase subunit B (SDHB)* are associated with malignancy and poor prognosis. When metastases arise, limited treatment options are available. The pathomechanism of *SDHB*-associated PPGL remains largely unknown, and the lack of suitable models hinders therapy development. Germline heterozygous *SDHB* pathogenic variants predispose to developing PPGLs with a life-long penetrance of around 50%. To mimic the human disease phenotype, we characterised adult heterozygous *sdhb* mutant zebrafish as a potential model to study *SDHB*-related PPGLs. Adult *sdhb* mutant zebrafish did not develop an obvious tumour phenotype and were anatomically and histologically like their wild-type siblings. However, *sdhb* mutants showed significantly increased succinate levels, a major hallmark of *SDHB*-related PPGLs. While basal activity was increased during day periods in mutants, mitochondrial complex activity and catecholamine metabolite levels were not significantly different. In conclusion, we characterised an adult in vivo zebrafish model, genetically resembling human carriers. Adult heterozygous *sdhb* mutants mimicked their human counterparts, showing systemic elevation of succinate levels despite the absence of a tumour phenotype. This model forms a promising basis for developing a full tumour phenotype and gaining knowledge of the pathomechanism behind *SDHB*-related PPGLs.

## 1. Introduction

Phaeochromocytomas and paragangliomas (PPGLs) are rare types of neuroendocrine cancers arising from chromaffin cells in the adrenal medulla or extra-adrenal paraganglia, respectively. In human patients with PPGLs of sympathetic origin, most signs and symptoms are due to the tumour’s catecholamine-secreting properties, resulting in paroxysmal palpitations, headache, hyperhidrosis, nausea, hypertension, and hyperglycaemia [1]. Approximately 40% of patients affected by PPGL are predisposed to this condition as a result of a heterozygous germline pathogenic variant in one of more than 20 identified susceptibility genes [2]. It is hypothesised that the acquisition of a second (somatic) mutation in addition to the existing germline mutation is one of the involved pathomechanisms of tumorigenesis [3]. Mutations in the various susceptibility genes are associated with distinct molecular and clinical phenotypes, and tumours can therefore be subdivided into three molecular clusters, with mutations causing (1) pseudohypoxic signalling, (2) aberrant kinase signalling, or (3) aberrant Wnt signalling [3]. Of all identified PPGL cases, 20% show mutations in one of the subunits of the *succinate dehydrogenase* (*SDH*) complex, leading to SDH deficiency and a pseudohypoxic phenotype. Mutations can occur in genes encoding one of four *SDH* subunits, *SDHA-D*, collectively referred to as *SDHx* mutations. Mutations in the gene *succinate dehydrogenase subunit B* (*SDHB*) are the most common and are associated with the highest malignancy rate. Other tumours associated with mutations in *SDHB* are renal cell carcinoma (RCC), gastrointestinal stromal tumour (GIST), and pituitary adenoma [4]. On average, the age of onset for *SDHB*-associated PPGLs is between thirty and fifty years old, but they may arise at any age [2]. When tumours arise, disease progression is monitored closely. In addition to universal monitoring techniques, such as imaging and symptom monitoring, disease (progression) in endocrine active tumours can be followed by the metabolisation of the tumour’s secreted catecholamines into metanephrines, measured in blood plasma or urine [5]. In the absence of metastases, management usually consists of surgical removal of the tumour. When metastases arise, in up to 50% of *SDHB*-related PPGL, treatment is challenging and is usually in a palliative setting [6].

*SDHB* encodes one of four subunits of the SDH complex, which is involved in the citric acid (TCA) cycle (converting succinate to fumarate) and the electron transport chain. Defects in SDH therefore lead to an accumulation of succinate and defective energy metabolism [7,8]. Succinate subsequently acts as an oncometabolite, altering the gene expression of genes involved in multiple pathways, including cell migration, cell invasion, and blood vessel formation [9]. Additionally, succinate inhibits demethylation, leading to epigenetic changes through genome hypermethylation [10].

The gaps in knowledge of the precise pathomechanism make it difficult to treat patients and develop new targeted therapies. Therefore, there is an urgent need to unravel the pathophysiological mechanisms behind *SDHB*-associated PPGL tumorigenesis.

To study the pathophysiology behind *SDHB*-associated PPGLs, a suitable model system is essential. Several model systems have been employed, from cell culture to canines, with varying levels of success [11]. In murine models, a homozygous mutation in *Sdhb* is embryonically lethal, whereas heterozygosity does not lead to a disease phenotype. Allografted mice models using *SDHB*-silenced cell lines showed tumour growth and developed metastases [12]. However, because of the genetic make-up of the allografted cells, their suitability as a disease model is limited. To date, the most accurate model system is a xenograft model, where PPGL cells derived from primary tumours in irradiated germline *Sdhb*-mutated rats were xenografted into immunocompromised mice. Resulting tumours recapitulated several human *SDHB*-associated PPGL characteristics, such as increased succinate levels, metabolic reprogramming, and transcriptional alterations related to defective electron transport and hypoxia [13]. These animals still lack natural tumour development following an *SDHB* mutation and therefore fail to recapitulate the human disease situation faithfully.

Recently, we proposed zebrafish as a model organism for *SDHB*-associated PPGLs. Generally, zebrafish have been established as an excellent cancer model system [14]. Apart from the obvious advantages of zebrafish as a model organism, such as transparency during early development, rapid development, and ease of inducing genetic alterations, there is a high degree of conservation of genes involved in cancer development, such as tumour suppressor genes, oncogenes, and cell-cycle genes. Moreover, their tumours are histologically similar to their human counterparts [14]. In the zebrafish inter-renal gland—the fish equivalent of the adrenal gland—chromaffin cells line the major blood vessels. Functionally, these chromaffin cells are homologous to mammalian adrenal medullary cells, synthesising and secreting catecholamines [15]. In addition, *sdhb* has been conserved in zebrafish and is a functional orthologue of human *SDHB* [16].

Previously, we developed a zebrafish model by CRISPR-Cas9 genome editing. In these zebrafish, homozygous *sdhb* mutant larvae showed human PPGL characteristics such as increased succinate levels and impaired energy metabolism, while their heterozygous and wild-type (WT) siblings showed a normal phenotype. However, homozygous larvae showed a severely decreased lifespan (up to 14 days), hindering possible observations of tumour formation in later life stages [16]. Therefore, the aim of the current study was to characterise the adult heterozygous *sdhb* mutant zebrafish. Adult mutants were characterised with respect to their behavioural phenotype, as well as anatomically, histologically, endocrinologically, and metabolically, to evaluate their suitability as a model for mimicking natural PPGL development in human carriers of pathogenic variants in *SDHB*.

## 2. Results

### 2.1. Lifespan and In Vivo Phenotypical Monitoring

**Figure 1:**
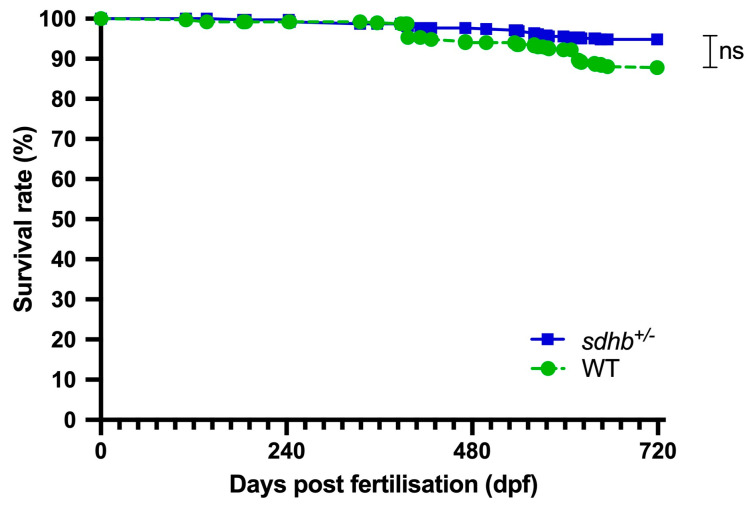
Monitoring of adult heterozygous *sdhb* mutant and WT zebrafish. Survival in heterozygous *sdhb* mutants and WT zebrafish. No significant difference (*p* > 0.05) was observed in the survival rate (%) between heterozygous *sdhb* mutants (n = 288) and WT zebrafish (n = 385). Statistical significance was tested with a log-rank (Mantel–Cox) test.

A total of 288 heterozygous *sdhb* mutant zebrafish and 385 WT zebrafish were monitored from the start of adulthood until death or a maximum of 2 years of age. No significant differences were observed in the 2-year survival rate (%) between heterozygous *sdhb* mutants (93%) and WT zebrafish (88%) (Figure 1). Deceased fish were either found dead, or a humane endpoint was applied. Of the observed heterozygous *sdhb* mutant zebrafish, 29 showed abnormalities during their lifespan, as opposed to two of their WT siblings (Figure S1). Five of those abnormalities were lumps, defined as abnormal growth, which were all identified in heterozygous *sdhb* mutant zebrafish of 1 year of age and older, without histological evidence of PPGL formation.

### 2.2. Behavioural Analyses

**Figure 2:**
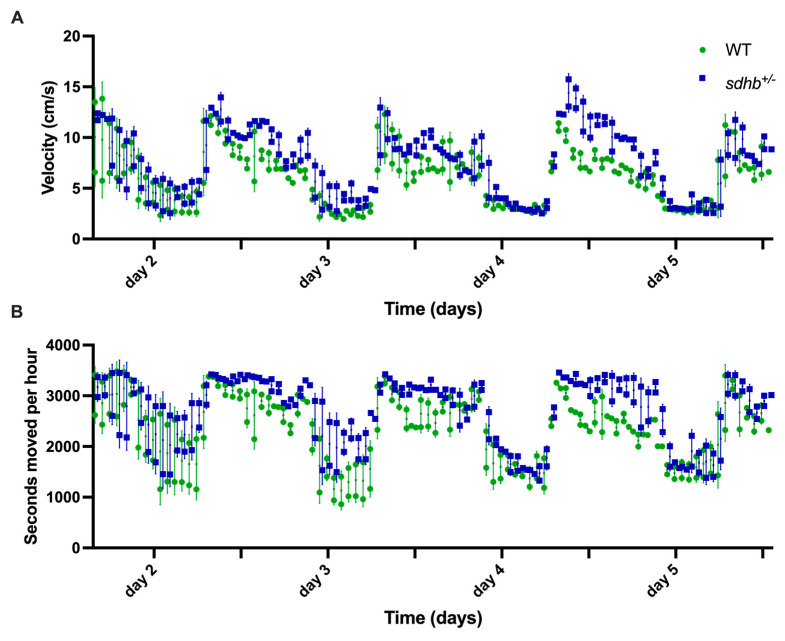
Behavioural analysis of adult heterozygous *sdhb* mutant (n = 5) and WT (n = 5) zebrafish. Swimming behaviour was tracked in two locomotor tests of 5 days. Ticks indicate 12-hour intervals. (**A**) Swimming velocity in centimetres per second. (**B**) Basal activity is displayed as cumulative seconds of movement. Basal activity was increased in heterozygous *sdhb* mutants during day periods (*p* < 0.05). There was no difference in swimming velocity between mutants and WT siblings during the day (*p* = 0.08) or night periods. The area under the curve (AUC) was used to evaluate statistical significance.

During general observations, no obvious alterations in swimming ability or behaviour were observed in heterozygous *sdhb* mutant zebrafish compared to their WT siblings. To investigate their swimming behaviour in more detail, 1.5-year-old fish were subjected to two locomotor tests for 5 days each. Basal activity, defined as seconds moved per hour, was significantly higher in heterozygous *sdhb* mutant zebrafish compared with their WT siblings during the day (*p* < 0.05) but not during the night. There were no differences in swimming velocity between WT and mutants during the day (*p* = 0.08) or night periods (Figure 2A,B).

### 2.3. Anatomy, Histology, and Chromaffin Cell Morphology

**Figure 3:**
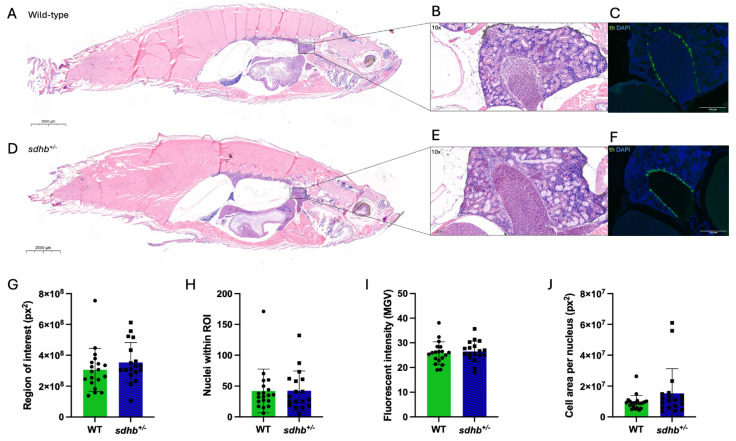
General histology, kidney morphology, and chromaffin cell morphology of adult heterozygous *sdhb* mutant and WT zebrafish. (**A**,**D**) General histology of adult heterozygous *sdhb* mutant (n = 34) and WT (n = 35) zebrafish (Representative images; H&E staining, 0.8× magnification). (**B**,**E**) Head kidney morphology (H&E staining, 10× magnification). (**C**,**F**) Immunohistochemical staining of tyrosine hydroxylase in the head kidney of heterozygous *sdhb* mutant (n = 10) and WT (n = 9) zebrafish (10× magnification). (**G**–**J**) Quantification of tyrosine hydroxylase staining (ImageJ2). Student’s *t* test was used to evaluate statistical significance. No significant differences were found in any of the analyses.

General anatomy, kidney morphology, and chromaffin cell morphology were analysed in heterozygous *sdhb* mutant and WT zebrafish (representative pictures shown in Figure 3A,B,D,E). H&E stainings of 34 heterozygous *sdhb* mutant zebrafish and 35 WT siblings were studied. No major overall anatomical differences were found between the heterozygous *sdhb* mutant and WT zebrafish. In addition, no differences in kidney morphology were observed between the two groups concerning tubular necrosis, tubular regeneration, haemorrhage of the interstitium, or tubular lumen enlargement. Ten heterozygous sdhb mutants and nine WT siblings were analysed in higher detail for chromaffin morphology (Figure 3C,F). No significant differences in the fluorescent area (region of interest; ROI), number of nuclei within that area, or fluorescence intensity were observed between the two groups (Figure 3G–I; Figure S2). Fluorescent area per nucleus was used as a proxy of cell size (Figure 3J, cell area per nucleus). There was a trend towards an increase in approximate cell size in heterozygous *sdhb* mutants (*p* = 0.0933).

### 2.4. Mitochondrial, Metabolomic, and Endocrine Phenotype

**Figure 4:**
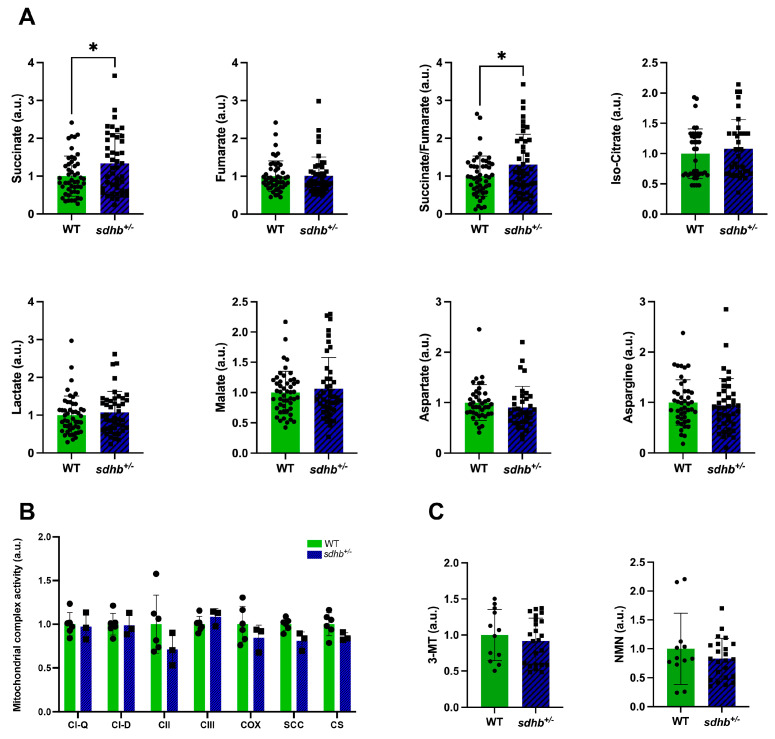
Metabolomics, mitochondrial complex activity, and metanephrine levels of adult heterozygous *sdhb* mutant and WT zebrafish. All values are standardised relative to the associated WT mean and expressed in arbitrary units (a.u.). (**A**) Abundance of metabolites in muscle tissue of heterozygous *sdhb* mutant (n = 44) and WT (n = 49) zebrafish (n is the total of three replicates). Succinate levels and succinate/fumarate ratios were significantly increased in heterozygous *sdhb* mutants compared with their WT siblings. (**B**) Mitochondrial complex activity in heterozygous *sdhb* mutant (n = 3) and WT (n = 6) zebrafish. Activity levels for all mitochondrial complexes were not significantly different. CI-D: complex I; CI-Q: quinone; CII: complex II; CIII: complex III; COX: complex V/cytochrome c oxidase; SCC: cytochrome P-450; CS: citrate synthase. (**C**) Catecholamine metabolite levels in the swimming water of heterozygous *sdhb* mutant (n = 23) and WT (n = 12) zebrafish. 3-MT: 3-Methoxytyramine; NMN: Normetanephrine. Student’s *t* test for metabolomics analysis and catecholamine metabolite analysis, Mann–Whitney U test for mitochondrial complex activity analysis. * *p* < 0.05.

Muscle tissue metabolite profiling showed significantly elevated succinate levels and succinate/fumarate ratio in heterozygous *sdhb* mutant zebrafish compared with WTs (*p* < 0.05, Figure 4A). No differences were observed for the other metabolites (fumarate, iso-citrate, lactate, malate, aspartate, and asparagine) measured. Comparing mitochondrial complex activities, no significant differences in complex II activity were observed in heterozygous *sdhb* mutant zebrafish (Figure 4B; *p* = 0.13). Lastly, analyses of catecholamine metabolites 3-methoxytyramine (3-MT) and normetanephrine (NMN) revealed no differences in 3-MT and NMN levels in fish water of heterozygous *sdhb* mutant and WT zebrafish (Figure 4C). Metanephrine levels were under the limit of detection and were therefore excluded from analysis.

## 3. Discussion

In this study, we characterised adult heterozygous *sdhb* mutant zebrafish to evaluate their potential as a model to study *SDHB*-related PPGLs. We showed that heterozygous mutants have a similar lifespan as their WT siblings. Mutant zebrafish were anatomically and histologically similar to their WT siblings. Behaviourally, *sdhb* mutant zebrafish showed higher basal activity compared with their WT siblings during day periods, with no differences in swimming velocity during day or night. The number of chromaffin cells in WT and heterozygous fish was similar, while there was a trend towards an increase in approximate cell size in heterozygous *sdhb* mutants. Moreover, there was no difference between heterozygous *sdhb* mutant and WT zebrafish in mitochondrial complex II activity. Notably, succinate levels were increased in the muscle tissue of heterozygous *sdhb* mutants. The remaining TCA cycle metabolites and mitochondrial complexes showed no differences in level or activity, nor did metanephrine(s) concentrations in swim water.

Genetically, our model represents the human *SDHB* carrier situation, possessing one mutated and one WT allele. In humans, mutations of *SDHB* are inherited in an autosomal dominant manner and portray incomplete penetrance, resulting in not all individuals with a pathogenic variant developing disease-related characteristics. Recent studies report 21% of *SDHB* carriers having developed at least one tumour at age 50, compared with 42% at age 70 [17]. Thus, of the currently published human *SDHB* mutation carriers, around half are expected to develop tumours during their lifetime. There are no known environmental risk factors for the development of PPGLs that explain why some carriers develop tumours and others do not.

During the two-year follow-up in this study, there were no differences in survival between heterozygous *sdhb* mutants and WT zebrafish. Of the limited number of abnormalities observed, there were five observations of abnormal growth, all observed in heterozygous *sdhb* mutants. We could provide no histological evidence of PPGL formation. While you could speculate that monitoring of the entire life span could provide us with more insight into possible natural PPGL development, it is questionable if tumours would arise at a later stage and if they would then be specifically PPGLs or solely age-related, let alone impractical to wait more than two years for natural tumour development. We expected a two-year time frame of study, which translates to 50 to 75% of the zebrafish lifespan, to be sufficient for tumour development, if this process were to occur naturally. Other germline-mutated zebrafish cancer models have been shown to develop tumours within twelve months of age, but these contain additional mutations to the mutation of interest, such as a knockout of p53 [18]. In addition to previously mentioned incomplete penetrance observed in humans, there are of course interspecies differences between zebrafish and humans that could explain the absence of tumour development. Moreover, laboratory animals are kept in sterile environments compared with regular human life, where we are exposed to all sorts of harmful environmental factors. It might thus be necessary to aggravate the genetic phenotype with treatments that accelerate the tumorigenesis process, such as SDH inhibitors.

A major hallmark of *SDHB*-related PPGLs is SDH deficiency and the ensuing accumulation of succinate. Succinate accumulation leads to the inhibition of α-ketoglutarate-dependent dioxygenases such as prolyl hydroxylase, which, under normoxic conditions, targets the transcription factor hypoxia-inducible factor for degradation. Prolyl hydroxylase (PHD) inhibition results in hypoxia inducible factor (HIF) stabilisation, inducing a pseudohypoxic response under normoxic conditions and altering the expression of genes involved in angiogenesis, cell migration, and cell invasion [9]. To investigate (early) signs of chromaffin cell hyperplasia, proliferation, or invasiveness, we analysed heterozygous *sdhb* mutant zebrafish and their WT siblings histologically. We observed no differences in chromaffin cell numbers or morphology between heterozygous *sdhb* mutant and WT zebrafish, providing no evidence of chromaffin cell hyperplasia or proliferation.

An important characteristic of PPGLs is the increased production and secretion of catecholamines. Zebrafish and mammalian endocrine systems are highly similar. While there are differences in endocrine gland structure, cellular processes are highly conserved. The endocrine function of chromaffin cells was histologically approximated by quantification of the staining intensity of tyrosine hydroxylase (th), a rate-limiting enzyme in the catecholamine synthesis pathway in chromaffin cells. However, we did not observe an increase in staining intensity in the chromaffin cells of heterozygous *sdhb* mutant zebrafish. In humans, TH is used solely as a marker for chromaffin cells. While, in our zebrafish model, we too use th as a marker for chromaffin cells, and staining intensity might also provide a proxy for transcript abundance and thus endocrine activity. This is reflected by studies in a rat-derived cell line for pheochromocytoma, where *Sdhb* silencing and consequent loss of complex II activity was associated with increased *Th* expression [19]. In addition, previous studies have shown that, in rats and a rat-derived PPGL cell line, (pseudo)hypoxia is associated with changes in *Th* mRNA levels and *Th* activity through altered phosphorylation [20,21]. The absence of differences in staining intensity in our model could possibly reflect that the systemic increase in succinate levels, measured in muscle tissue of heterozygous *sdhb* mutants in the presence of sustained complex II activity, is insufficient to drive altered chromaffin cell morphology or endocrine function, and additional triggers are necessary.

A limitation of this quantification is the high variability between histological sections because of variations in the sectioning plane. This causes high variation in the visualisation of chromaffin cells, complicating the quantification of chromaffin cells. However, histological analysis does allow us to observe tumour development when any arise. A transgenic line, fluorescently labelling chromaffin cells, could provide a solution for this, allowing for their isolation via fluorescence-activated cell sorting (FACS). Next to the exact quantification of chromaffin cell numbers, this would enable additional analyses such as gene expression analyses by quantitative PCR or methylation assays. The high variability in the histological sections now forces us to look at group averages, while interindividual differences are large, also because of the aforementioned incomplete genetic penetrance. Moreover, looking at the individual would allow for the correlation of multiple measurements, such as relating chromaffin cell functionality to succinate levels.

To reliably assess the endocrine function of chromaffin cells, catecholamine and metanephrine levels were assessed, the latter being the metabolised product of the former. Of note is that while the methodology to measure catecholamines and their metabolites in blood plasma, urine, and tissue extracts is well established [22], we are, to our knowledge, the first to measure excreted catecholamines and their metabolic by-products in fish water. In patients, most signs and symptoms are due to the tumour’s catecholamine-secreting properties. In agreement with our histological analyses, we observed no differences in metanephrine levels, probably reflecting the lack of difference in chromaffin cell mass between heterozygous *sdhb* mutant and WT zebrafish.

SDH deficiency and succinate accumulation in *SDHB*-related PPGLs is accompanied by a decrease in other TCA cycle metabolites, including fumarate, cis-aconitate, and isocitrate [23]. Consequently, an increased succinate/fumarate ratio is used as a metabolic marker to diagnose *SDHB*-related PPGLs [24]. Interestingly, we found a significant increase in succinate and the succinate/fumarate ratio in heterozygous *sdhb* mutants compared with WT zebrafish, with no differences in other TCA cycle metabolites, indicating at least impaired SDH enzymatic activity despite the presence of a WT allele. In this aspect, again, this model is a faithful and unique representation of the human *SDHB* carrier situation, as *SDHx* mutation carriers often have elevated succinate levels in the absence of tumours or other symptoms [25,26]. This is as opposed to complete homozygosity leading to complete SDH deficiency and a clear metabolic phenotype, such as in Leigh syndrome [27].

The presumed residual SDH activity is reflected by the mitochondrial complex activity. We observed no significant differences between heterozygous mutants and WT zebrafish measured in muscle tissue. This can be most likely attributed to the functional copy present in heterozygous mutants but might be partly attributed to the analysed tissue type or small sample size. This agrees with earlier observations in the larval stages of this model, where there was almost complete loss of complex II activity in homozygous *sdhb* mutant larvae but no decrease in heterozygous mutants [16]. Again, our model reflects the human *SDHB* carrier situation by portraying elevated succinate levels in the absence of a decrease in complex II activity.

Impaired energy metabolism is another characteristic of *SDHB*-related PPGLs because of the role of SDH in the electron transport chain. *SDHx*-mutated tumours have been shown to have impaired ATP production, increased activity of complexes I, III, and IV, and an increased number of morphologically and functionally abnormal mitochondria [7]. Indeed, this was reflected in the larval stages of this model, where energy metabolism, illustrated by swimming behaviour, was shown to be impaired in homozygous *sdhb* mutant larvae with no differences in swimming behaviour between heterozygous *sdhb* mutant and WT larvae [16]. In addition, succinate has been reported to be involved in energy homeostasis through succinate receptor 1 (SUCNR1) signalling [28,29]. We showed that, upon ageing, succinate accumulates in heterozygous mutants. These mutants also show increased basal activity during day periods, with no differences in swimming velocity between mutant and WT zebrafish, neither during the day nor the night periods. This difference in behaviour cannot be solely attributed to energy metabolism but could be the cumulative result of many additional factors, such as anxiety or stress levels.

We were unable to discern evidence of PPGL tumour development in adult heterozygous *sdhb* mutant zebrafish. In humans, a second hit is often necessary to develop PPGLs [3]. This is reflected in mice models, where systemic *Sdhb* heterozygosity is insufficient to induce tumour formation [10,11], *Sdhb* knock-out in medullary adrenal cells solely led to increased succinate levels in the absence of other PPGL hallmarks, but additional *Nf1* mutations induced *SDHx*-like PPGLs [30]. The introduction of additional mutations could be beneficial to induce tumour formation in our model. However, the molecular genetics underlying PPGL development are complex, resulting in different molecular tumour subtypes for different pathogenic variants [3]. Therefore, to faithfully recapitulate the human disease situation, it is crucial to reflect the patient situation genetically. Compared with the above-mentioned animal models for *SDHB*-related PPGLs, our model is the first adult model to recapitulate the relevant genetic background faithfully while partially recapitulating a disease phenotype. Genetically and metabolically, our model represents the human *SDHB* carrier situation, where heterozygosity is associated with an increase in succinate levels in the absence of any other symptoms [23]. Although no natural tumour development has been confirmed as of yet, this model forms a solid base for future research, where we aim to aggravate the current phenotype by altering the involved signalling pathways or inducing additional mutations in a tissue-specific manner. The current absence of natural tumour development allows for the investigation of the trigger necessary for tumour initiation when a heterozygous *SDHB* pathogenic variant is already present.

In conclusion, we characterised an adult in vivo zebrafish model for *SDHB*-related PPGLs, showing a partial disease phenotype through elevated succinate levels. However, increased succinate could not be correlated to other phenotypic abnormalities, indicating that *sdhb* heterozygosity in itself is insufficient to cause a full PPGL phenotype. While the current model does not allow for investigating tumour initiation and development, it is a unique model representing the human carrier situation genetically and metabolically, enabling research into risk factors associated with *SDHB* heterozygosity. In future studies, additional mutations to stimulate a second hit might be necessary to induce a full disease phenotype. In addition, we intend to aggravate the current partial phenotype by altering involved pathways, thereby hopefully identifying the trigger necessary for tumour formation. Altogether, we present an adult zebrafish model with promising prospects to gain knowledge of the pathomechanism behind *SDHB*-related PPGLs.

## 4. Materials and Methods

### 4.1. Zebrafish Husbandry

Experimental procedures were conducted in accordance with institutional guidelines and National and European legislation. Ethical approval of the experiments was granted by Radboud University’s Institutional Animal Care and Use Committee (IACUC, application number RU-DEC 2020-0030). A germline heterozygous *sdhb* mutation was introduced in zebrafish larvae (*Danio rerio*), in an Oregon AB background, through CRISPR-Cas9 genome editing by our group previously (*sdhb^rmc200^*; ZFIN: ZDB-FISH-220110-2; [16]). This resulted in zebrafish harbouring a 13-base pair deletion at the exon1-intron1 boundary, leading to a frameshift. Heterozygosity of the mutant allele or WT *sdhb* was confirmed through PCR amplification. Eggs were obtained from natural spawnings. Larvae were maintained and raised by standard methods [31].

### 4.2. Genotyping

Adult zebrafish were briefly anaesthetised in 2-phenoxyethanol (0.1%, *v*/*v*). To obtain genomic DNA, a small section of the tail fin was cut using microscissors. The sample was placed in 25 µL lysis buffer (40 mM NaOH, 0.2 mM EDTA), followed by incubation at 95 °C for 30 min. After incubation, samples were neutralised by adding 75 µL 0.33 mM Tris-HCl. The extracted genomic DNA was used as a PCR template. Primer sequences used to amplify *sdhb* exon 1 were 5′-CATGGCGGCTGTGTGTTTCT-3′ (fw) and 5′-ACGGGTTATTGATGAGCGGTG-3′ (rv). As input, 15.1 µL water, 0.2 µL Dreamtaq (5 U/µL, Thermo Scientific™, Waltham, MA, USA), 0.5 µL dNTPs (10 µM), 1 µL fw primer (10 µM), 1µL rv primer (10 µM), 2 µL PCR buffer, and 1 µL of genomic DNA were used. The cycling conditions were as follows: 94 °C 2 min, 35 cycles of 94 °C 15 s, 58 °C 30 s, 72 °C 30 s, and 72 °C 2 min. The obtained amplicons (WT: 123 base pairs (bps); heterozygous *sdhb* mutant: 123 bp and 110 bp) were visualised on a 2% Tris-borate-EDTA (TBE) agarose gel.

### 4.3. Behavioural Assessment

Using EthoVision XT7 (Noldus Information Technologies, Wageningen, The Netherlands), the locomotion of adult zebrafish aged 1 to 1.5 years old was tracked. This study was conducted using five heterozygous *sdhb* mutant zebrafish and five WT siblings, placed in two separate aquaria (15 × 30 cm) on an infrared light source, equipped with an Area scan camera (Basler, Ahrensburg, Germany, 106588). The zebrafish were tracked for 5 days. Tracking was performed twice with the same batch of fish. Cumulative time moved (s) and velocity (cm/s) were used as read-outs for behaviour. The data were analysed using Graphpad Prism software (Version 10.1.1 for macOS).

### 4.4. Histology and Immunohistochemistry

Adult zebrafish were euthanized using an overdose of 2-phenoxyethanol (0.1% *v*/*v*). After euthanasia, a ventral incision was made to ensure complete fixation. Fish were fixated in 4% paraformaldehyde (Sigma, Saint Louis, MO, USA) overnight and subsequently rinsed in tap water (1 h) before a 3-day decalcification in Mol-DECALCIFIER (Menarini Diagnostics, Florence, Italy). After another tap water rinse (1 h), the tissue was kept in 4% paraformaldehyde for sample transfer to tissue processing. Tissue processing from paraformaldehyde to paraffin was performed using the overnight MAGNUS protocol as follows: after a 60-min fixation at 37 °C and a 70 min fixation at 50 °C, samples were subsequently incubated in ethanol (60 min, 55 °C), isopropanol (110 min, 55 °C), and finally, isopropanol (245 min, 65 °C). Vaporisation was performed at 500 mBar (1.5 min), after which the samples were impregnated with wax in a 7-step impregnation series as follows: (1) 25 min, 500 mBar, 70 °C, (2) 15 min, 400 mBar, 70 °C, (3) 10 min, 300 mBar, 70 °C, (4) 10 min, 200 mBar, 70 °C, (5) 10 min, 150 mBar, 70 °C, (6) 150 min, 100 mBar, 65 °C, and (7) 20 min, 800 mBar, 65 °C.

To assess morphology, paraffin sections (5 μm) were mounted on SUPERFROST^®^ PLUS Menzel-Gläser (Thermo Scientific™, Waltham, MA, USA) slides and dried overnight at 37 °C. The slides were stained with haematoxylin and eosin as described previously [32] and imaged with a Zeiss Axioskop (Zeiss, Oberkochen, Germany) light microscope.

To visualise chromaffin cells for the detection of PPGLs and/or hyperplasia, selected slides based on correct morphology were stained for tyrosine hydroxylase (TH), the rate-limiting enzyme in the catecholamine synthesis pathway. Selected sections were deparaffinized with xylene (2 × 10 min), followed by rehydration in decreasing ethanol dilutions (96, 90, 80, 70 and 50%, 3 min each), demineralised water (3 min), and phosphate-buffered saline (PBS). After antigen retrieval (10 mM Citrate buffer, pH 6.0), the slides were blocked with 2% bovine serum albumin (BSA) in PBS for 1 h at room temperature (RT). The slides were washed with PBS (3 × 5 min) and incubated with Anti-Tyrosine Hydroxylase Antibody (Merck, Rahway, NJ, USA, AB1542; 1:1000 in 2% BSA in PBS) overnight at 4 °C. The slides were then washed again with PBS (3 × 5 min) before incubation with the secondary antibody (Alexa Fluor 488 Donkey Anti-Sheep; Thermo Fisher Scientific, Waltham, MA, USA; 1:1000 in 2% BSA in PBS) for 1 h at RT in the dark. From there on, the slides were kept in a dark environment. After washing with PBS (3 × 5 min), the slides were counterstained with Sudan Black (0.1% in 70% ethanol) for 10 min at RT. After washing with PBS (3 × 5 min), the slides were coverslipped using DAPI-Fluoromount-G (ITK Diagnostics, Uithoorn, The Netherlands) and dried overnight at RT before imaging head kidney regions, containing chromaffin cells, and other regions of interest. Imaging was performed using a Zeiss Axio Imager (M1) (Zeiss, Oberkochen, Germany) equipped with an AxioCam MRm.

Image analysis was performed using FIJI software (ImageJ2, version 2.12.0/1.54f), excluding images of an incorrect anatomical location or containing major staining artefacts affecting downstream image analysis. An automated script was generated to perform objective quantitative image analysis (10.6084/m9.figshare.25522921). The fluorescent area (region of interest; ROI), number of nuclei in the fluorescent area (maxima), and staining intensity of the fluorescent area (mean grey value; MGV) were used as read-outs for chromaffin cell morphology.

### 4.5. Mitochondrial Complex Activity Measurements

Mitochondrial complex activity levels were measured in the muscle tissue of WT and heterozygous *sdhb* mutant adult zebrafish. After euthanasia, approximately 10 mg of muscle tissue was taken from the tail of the zebrafish. The samples were weighed, snap-frozen in liquid N2, and stored at −80 °C. Defrosted material was homogenised in 1450 μL 10 mM Tris-HCl, pH 7.6. Then, 250 μL 1.5 M sucrose was added, followed by 10 min 14,000× *g* centrifugation at 2 °C. The supernatant was discarded, and the pellet was resuspended in 200 μL 10 mM Tris-HCl, pH 7.6. The enzymatic activities of OXPHOS complexes I to V, citrate synthase, and the sample protein content were assayed spectrophotometrically as previously described [33]. Assays were performed in duplicate using a Konelab 20XT auto-analyser (Thermo Fisher Scientific, Waltham, MA, USA).

### 4.6. Mass Spectrometry of TCA Cycle Metabolites and Amino Acids

Using a mass spectrometry-based assay, metabolites involved in the TCA cycle were measured in the muscle tissue of WT and heterozygous *sdhb* mutant adult zebrafish. After euthanasia, approximately 10 mg of muscle tissue was taken from the tail of the zebrafish. The samples were weighed, homogenised in 100 μL methanol together with an internal standard mixture, centrifuged to precipitate insolubles, and dried in a speed vac concentrator. Metabolite extracts were resuspended in the mobile phase and analysed by liquid chromatography with tandem mass spectrometry (LC-MS/MS) as described elsewhere [34]. Data were normalised based on weight.

### 4.7. Metanephrine Measurements

To detect PPGL-related catecholamine excess, catecholamine metabolite levels were measured in the fish water of WT and heterozygous *sdhb* mutant adult zebrafish. Zebrafish were individually housed in 500 mL water for three days before sample collection. The samples were snap-frozen in liquid nitrogen and stored at −80 °C until analysis. Catecholamine metabolite levels were measured in fish water after solid-phase extraction (SPE) of 1 mL sample volume using a liquid chromatography–mass spectrometry (LCMS) based method, as described previously [35].

### 4.8. Statistical Analysis

Graphpad Prism software (Version 10.1.1 for macOS) was used to generate scatter plots, calculate mean values, and perform statistical analyses. The data were tested for Gaussian distribution. The log-rank (Mantel–Cox) test was used for the survival curve analysis, student’s *t* test for metabolomics analyses, catecholamine metabolite analysis, and immunohistochemical staining quantification, and the Mann-Whitney U test for mitochondrial complex activity analyses. Behavioural data were statistically tested using area under the curve (AUC) analysis. R software (R 4.3.2 GUI 1.80 Big Sur ARM build (8281)) was used to generate density plots.

## Data Availability

The raw data supporting the conclusions of this article will be made available by the authors upon request.

## Supplementary Materials

The following supporting information can be downloaded at https://www.mdpi.com/article/10.3390/ijms25137262/s1.
