# Supplementary material for: Characterisation of an Adult Zebrafish Model for SDHB-Associated Phaeochromocytomas and Paragangliomas
Source: Int J Mol Sci. 2024 Jul 1;25(13):7262. doi: 10.3390/ijms25137262 (PMC11241774; doi:10.3390/ijms25137262)
Supplement: Supplementary file 1 [file ijms-25-07262-s001.zip › ijms-3055327-supplementary.pdf]

Supplementary

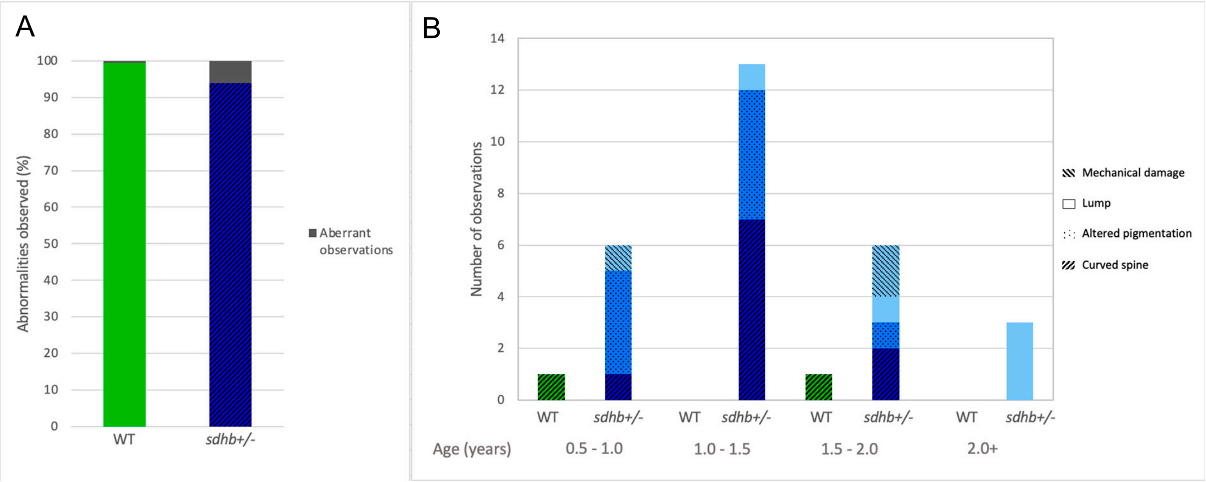

Figure S1. Long-term *in vivo* phenotypical monitoring of adult heterozygous *sdhb* mutant and WT zebrafish. (A) Heterozygous *sdhb* mutant (*n* = 288) and WT (*n* = 385) were followed up to 2 years of age for the occurrence of external abnormalities. **(B)** Noted abnormalities were classified into curved spine, altered pigmentation, mechanical damage or lumps. We could provide no evidence of PPGL tumour formation.

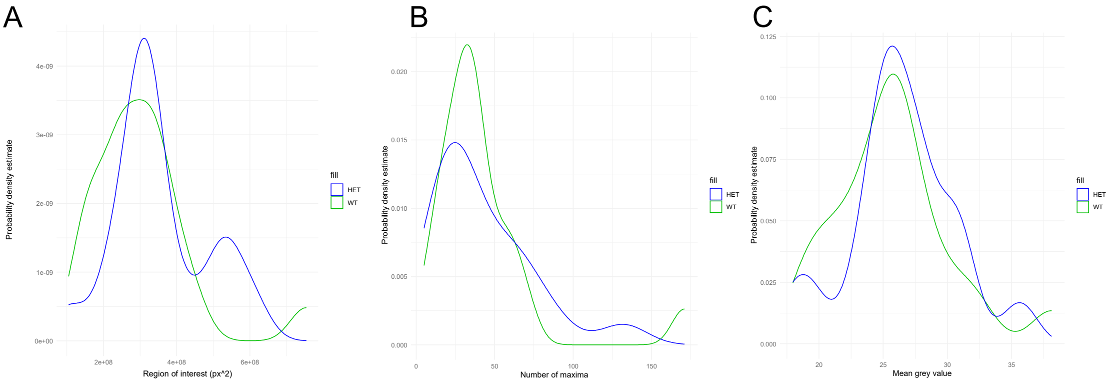

Figure S2. Distribution of chromaffin cell quantification of adult heterozygous *sdhb* mutant and WT zebrafish. Chromaffin cells of heterozygous *sdhb* mutant (*n* = 10) and WT (*n* = 9) zebrafish were analysed with ImageJ2 after immunohistochemical staining of tyrosine hydroxylase. Quantification read-outs were **(A)** fluorescent area (region of interest; ROI), **(B)** number of nuclei within ROI and **(C)** fluorescent intensity within ROI (Mean Grey Value; MGV). The probability density estimate portrays the relationship between the observed value on the x-axis and its probability. Density plots were created using R software (R 4.3.2 GUI 1.80 Big Sur ARM build (8281)).
